# Cmpd10357 to treat B-cell acute lymphoblastic leukemia

**DOI:** 10.1016/j.exphem.2022.12.005

**Published:** 2023-01-06

**Authors:** Alex Q. Lee, Hiroaki Konishi, Elizabeth Helmke, Masami Ijiri, Jan Michael A. Lerot, Emma Hicks, Jeremy R. Chien, Fredric A. Gorin, Noriko Satake

**Affiliations:** a Department of Pediatrics, UC Davis School of Medicine, Sacramento, CA; b Department of Biochemistry and Molecular Medicine, UC Davis School of Medicine, Sacramento, CA; c Department of Neurology, UC Davis School of Medicine, Sacramento, CA; d Department of Molecular Biosciences, UC Davis School of Veterinary Medicine, CA

## Abstract

B-cell acute lymphoblastic leukemia (B-ALL) is the most common type of cancer found in children. Although the overall survival rates are now >80%, 15%–20% of pediatric patients relapse, with survival rates subsequently dropping to 5%–10%. Cmpd10357, 3-amino-5-arylamino-6-chloro-N- (diaminomethylene) pyrazine-2-carboximide, is a highly potent, cell-permeant compound recently shown to have cytotoxic effects on solid tumors, including human breast cancer and high-grade gliomas, independent of their proliferative status. Cmpd10357 demonstrated concentration-dependent cytotoxicity in two human B-ALL cell lines, JM1 and Reh, at half-maximal inhibitory concentrations (IC_50_) of 3.2 and 3.3 *μ*M, respectively. Cmpd10357, at a dose of 5 mg/kg, significantly prolonged survival in our B-ALL xenograft mouse model, with a median survival time of 49.0 days compared with 45.5 days in the control group (*p* < 0.05). The cytotoxicity of Cmpd10357 demonstrated caspase-independent, nonapoptotic cancer cell demise associated with the nuclear translocation of apoptosis-inducing factor (AIF). The cytotoxicity of Cmpd10357 in B-ALL cells was inhibited by Necrostatin-1 but not by Necrosulfonamide. These studies suggest that an AIF-mediated, caspase-independent necrosis mechanism of Cmpd10357 in B-ALL could be used in combination with traditional apoptotic chemotherapeutic agents

B-cell type acute lymphoblastic leukemia (B-ALL) is the most common type of cancer found in children. Although survival rates for B-ALL are >80% largely owing to the development of combination chemotherapy regimens, relapse occurs in 15%–20% of pediatric patients, and survival rates after relapse can drop to 5%–10% [1–4]. Therefore, novel therapeutic approaches are needed.

Cmpd10357 is a substituted pyrazine-2-carboximide compound that has demonstrated its cytotoxicity in glioma [5,6]. In this study, we illustrate for the first time the therapeutic potential of Cmpd10357 for B-ALL both in vitro and in vivo. We also confirm the nonapoptotic necrosis mechanism of Cmpd10357 in B-ALL cells involving nuclear translocation of apoptosis-inducing factor (AIF), without activation of caspase 3/7, endoplasmic reticulum (ER) stress, or mixed lineage kinase domain-like protein (MLKL)-mediated necroptosis. These findings suggest a potential synergistic role of Cmpd10357 in combination with current apoptotic chemotherapeutic agents for the treatment of resistant and relapsed B-ALL cancer subtypes.

## METHODS

### Reagents and Drugs

**Figure 1:**
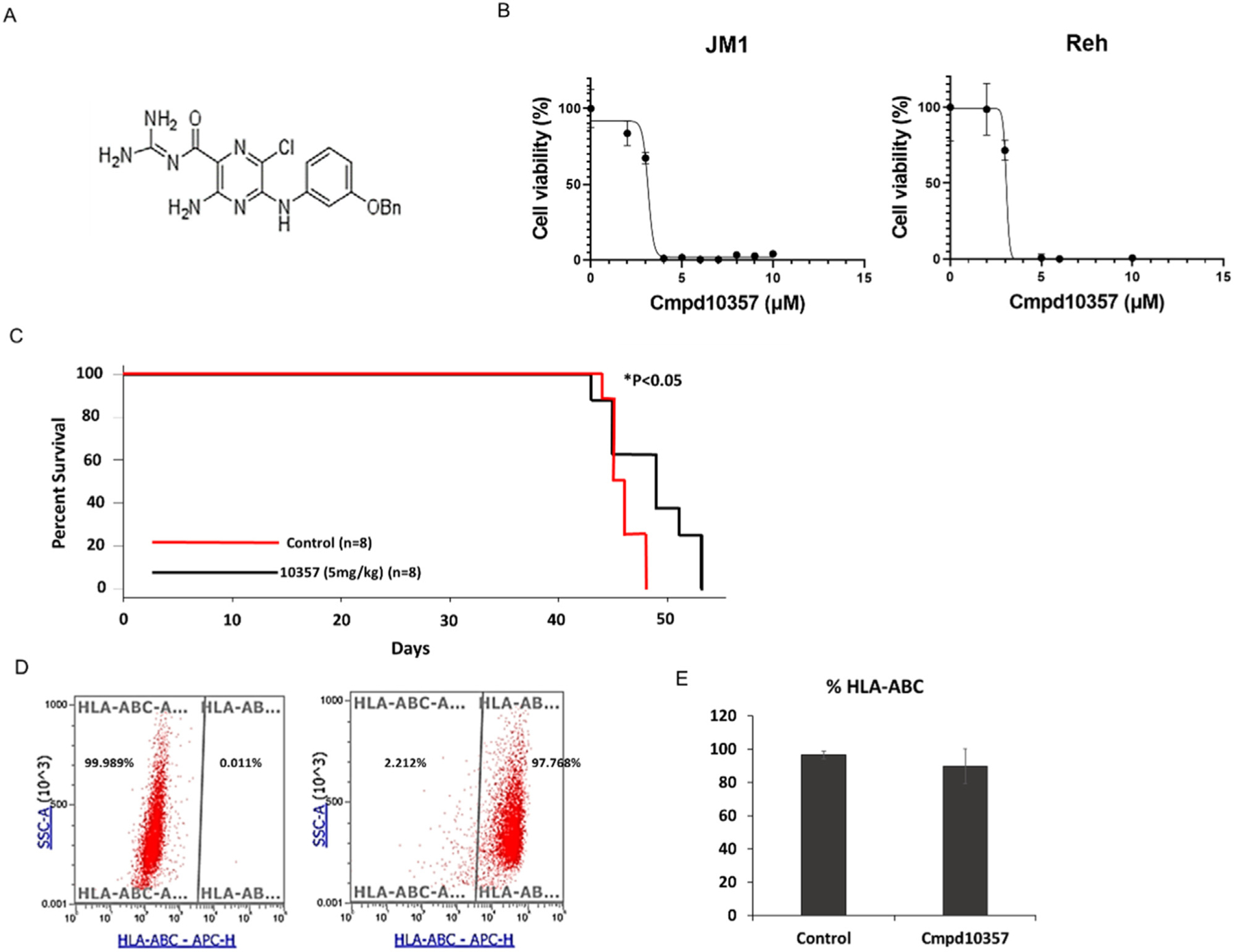
Cytotoxicity of Cmpd10357 in B-ALL cell lines and the B-ALL mouse xenograft model. (**A**) The chemical structure of Cmpd10357 is shown. (**B**) Cmpd10357 shows cytotoxicity in both JM1 (left) and Reh (right), with IC_50_ of 3.2 and 3.3 *μ*M, respectively. (**C**) Cmpd10357 shows significant in vivo therapeutic efficacy and prolongs survival time in a JM1 human leukemia mouse model (*p < 0.05). A Kaplan-Meier survival curve for mice inoculated with JM1 cells and treated with Cmpd10357 is shown. Mice were treated with 80% PEG or 5-mg/kg Cmpd10357. (**D**) Harvested cells from the bone marrow upon euthanasia were nearly 100% HLA-positive by flow cytometry. (**E**) The average of HLA-positive cells from the bone marrow is shown. Means ± SDs are presented. APC-H=allophycocyanin-H; SSC-A=side scatter-A.

Cmpd10357, 3-amino-5-arylamino-6-chloro-N-(diaminomethylene) pyrazine-2-carboximide, was provided by Fredric Gorin’s and Pamela Lein’s research unit (Figure 1A) [6]. Cmpd10357 was reconstituted in dimethyl sulfoxide (DMSO) to a final concentration of 250 mM and stored at −20°C.

The receptor-interacting serine/threonine-protein kinase 1 (RIPK1) inhibitor, Necrostatin-1 (Thermo Fisher Scientific), or the MLKL inhibitor, Necrosulphonamide (Cayman Chemical), was added to culture media at 30 and 2 *μ*M, respectively, on the basis of a similar study [7,8] with Cmpd10357.

### Cell Lines

Two human precursor B-ALL cell lines, JM1 (https://www.cellosaurus.org/CVCL_3532) and Reh (https://www.cellosaurus.org/CVCL_1650), were purchased from ATCC. Cells were maintained, grown, and counted as described in the Supplementary Methods.

### MTS (3-(4,5-dimethylthiazol-2-yl)-5-(3-carboxymethoxyphenyl)-2-(4-sulfophenyl)-2H-tetrazolium) Assay

JM1 or Reh cells were seeded in 96-well plates and treated with drugs. 3-(4,5-dimethylthiazol-2-yl)-5-(3-carboxymethoxyphenyl)-2-(4-sulfophenyl)-2H-tetrazolium (MTS) assays were performed as described in the Supplementary Methods.

### Caspase-Glo 3/7 Assay

Cells were treated with Cmpd10357 or hydrogen peroxide (H_2_O_2_), and caspase activity was measured using a Caspase-Glo 3/7 assay kit (Promega) according to the manufacturer’s instructions.

### Leukemia Cell Transplantation

The JM1 leukemia mouse model was created by intratibial injection of 2 × 10^6^ cells into both tibias of healthy 14–17-week-old male Non-obese diabetic, severe combined immune deficiency, interleukin 2 receptor, gamma chain (NSG) mice using our institutionally approved animal care protocol.

### Drug Efficacy Study

Cmpd10357 was compared with a vehicle control for overall survival in JM1 leukemia model mice. Sixteen mice injected with 2 × 10^6^ JM1 cells per mouse were randomly enrolled into two groups: group 1, vehicle control with 80% polyethylene glycol 400 (PEG) (Spectrum Chemical Mfg. Corp.) in phosphate-buffered saline (PBS) (n = 8), and group 2, Cmpd10357 (n = 8). The vehicle control group was dosed by daily intraperitoneal (IP) administration for 2 weeks. A 250-mM Cmpd10357 stock solution was dissolved in 80% PEG and administered intraperitoneally daily at 5 mg/kg for 2 weeks. Mice were monitored daily and euthanized when they showed signs of leukemia in accordance with Institutional Animal Care and Use Committee (IACUC) policy on humane end points. Leukemia cells were harvested from the leukemia-infiltrated bone marrow for experiments. JM1 leukemia cells were confirmed by flow cytometry using an anti-human leukocyte antigen (HLA)-ABC antibody (Biolegends and BD Biosciences) for phenotyping. Statistical significance for survival time was determined by the log-rank test. Kaplan-Meier survival curves were plotted for the two groups. Analyses were performed using Prism 8.3 software (GraphPad).

### Immunoblot Analysis

For total cell protein extractions, cell pellets from 1–2 × 10^6^ cells were denatured in the radioimmunoprecipitation assay (RIPA) lysis buffer (VWR Life Science). For nuclear fractions, cell pellets from 3–5 × 10^6^ cells were denatured using NE-PER Nuclear and Cytoplasmic Extraction Reagents (Thermo Fisher Scientific), and nuclear fractions were collected according to the manufacturer’s instructions. Immunoblotting and analysis were performed as described in Supplementary Methods.

## RESULTS AND DISCUSSION

### Cmpd10357 Demonstrates Cytotoxicity in B-ALL Cell Lines

In this study, we used two well-characterized B-ALL cell lines, JM1 and Reh. To evaluate the in vitro cytotoxicity of Cmpd10357 in B-ALL cell lines, JM1 and Reh cells were treated with Cmpd10357 for 72 hours (n = 3). Cell viability was measured using the MTS assay. Cmpd10357 demonstrated concentration-dependent cytotoxicity in both JM1 and Reh with half-maximal inhibitory concentrations (IC_50_) of 3.2 and 3.3 *μ*M, respectively (Figure 1B).

### Cmpd10357 Improves Survival in a B-ALL Xenograft Mouse Model

Having demonstrated the anti-cancer effects of Cmpd10357 on B-ALL in vitro, we created xenograft mouse models using JM1 cells. First, a dose identification study was performed with 2.5, 5, and 10 mg/kg of Cmpd10357 based on the previous study reporting 10 mg/kg Cmpd10357 as an effective and well-tolerated dose in glioma models [5]. Because the doses of 5 mg/kg and 10 mg/kg of Cmpd10357 did not show any differences in efficacy, we conducted the therapeutic study using a 5-mg/kg dose. The median survival time of the Cmpd10357-treated group was significantly longer than that of the vehicle control group, 49.0 days versus 45.5 days (*p* < 0.05) (Figure 1C). Upon euthanasia, human leukemia (JM1) was confirmed by HLA class I (HLA-ABC) positivity of harvested bone marrow cells of mice by flow cytometry. Most of the mice had nearly 100% human leukemia in their bone marrow (Figure 1D and E). No mice perished while Cmpd10357 was administered. Bowel obstruction was observed in two mice in the vehicle control group and in one mouse in the Cmpd10357 group, and diarrhea was observed in two mice in the Cmpd10357 group. These toxicities could be due to high concentration of PEG; however, further studies with larger cohorts will be required to confirm the safety profile of Cmpd10357.

### Cmpd10357 Induces Cytotoxicity Through Nonapoptotic Cell Death in B-ALL Cells

**Figure 2:**
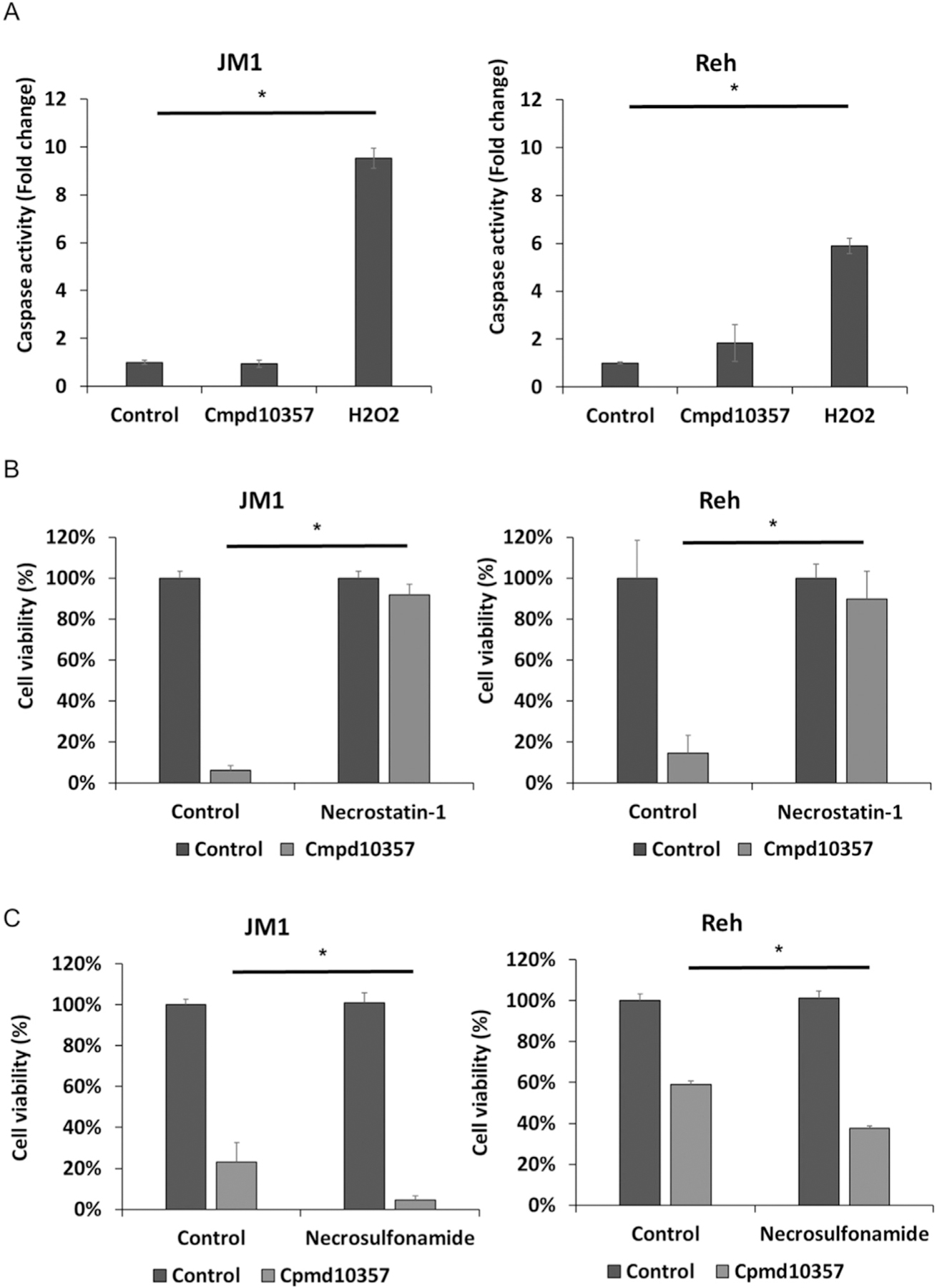
Cmpd10357 induces cytotoxicity through nonapoptotic cell death mediated by RIPK1 in JM1 and Reh cells. (**A**) Caspase-Glo 3/7 assays showed that 5-*μ*M Cmpd10357 treatment did not increase caspase activity, whereas 400-*μ*M H_2_O_2_ treatment did (n = 3). MTS assay showed that cytotoxicity of 5-*μ*M Cmpd10357 is reversed in the presence of (**B**) 30-*μ*M Necrostatin-1 (n = 3) but not in the presence of (**C**) 2-*μ*M Necrosulfonamide (n = 3). All data are presented as the mean ± SD. *p < 0.05.

To investigate the mechanism of Cmpd10357-induced cytotoxicity, caspase-mediated apoptosis was assessed in JM1 and Reh cells. Caspase-Glo 3/7 assays showed that Cmpd10357 did not induce significant caspase activity in JM1 and Reh cells, compared with H_2_O_2_ control (Figure 2A). We also assessed that ER stress–mediated cell death pathway as another substituted pyrazine derivative, 5-benzylglycinyl amiloride (UCD38B) binds with intracellular uPA-PAI-1 complexes and is taken up by the ER, causing ER stress in glioma cells [5]. Cell fractions harvested at 6, 8, and 24 hours of the treatment were immunoblotted for ER stress markers activating transcription factor 4 (ATF4), phosphorylated eukaryotic initiation factor 2 (P-eIF2), and C/EBP-homologous protein (CHOP) and for actin as a total protein marker. None of these three marker expressions, except P-eIF2a in JM1 at 24 hours, were significantly increased (quantified results at each time point are shown in Supplementary Figure E1). These results suggest that Cmpd10357 did not induce caspase or ER stress–mediated cell death in B-ALL cells.

To further investigate the mechanism of Cmpd10357’s cytotoxicity, JM1 and Reh cells were treated with Cmpd10357 in the presence or absence of the RIPK1 inhibitor, Necostatin-1. MTS assays demonstrated that Necrostatin-1 significantly reversed the viability of Cmpd10357-treated JM1 and Reh cells (Figure 2B). On the other hand, a MLKL inhibitor, Necrosulfonamide, did not reverse the viability of Cmpd10357-treated JM1 and Reh cells (Figure 2C), suggesting RIPK1-mediated nonapoptotic cell death.

### Cmpd10357-Induced Cytotoxicity Involves AIF Translocation

**Figure 3:**
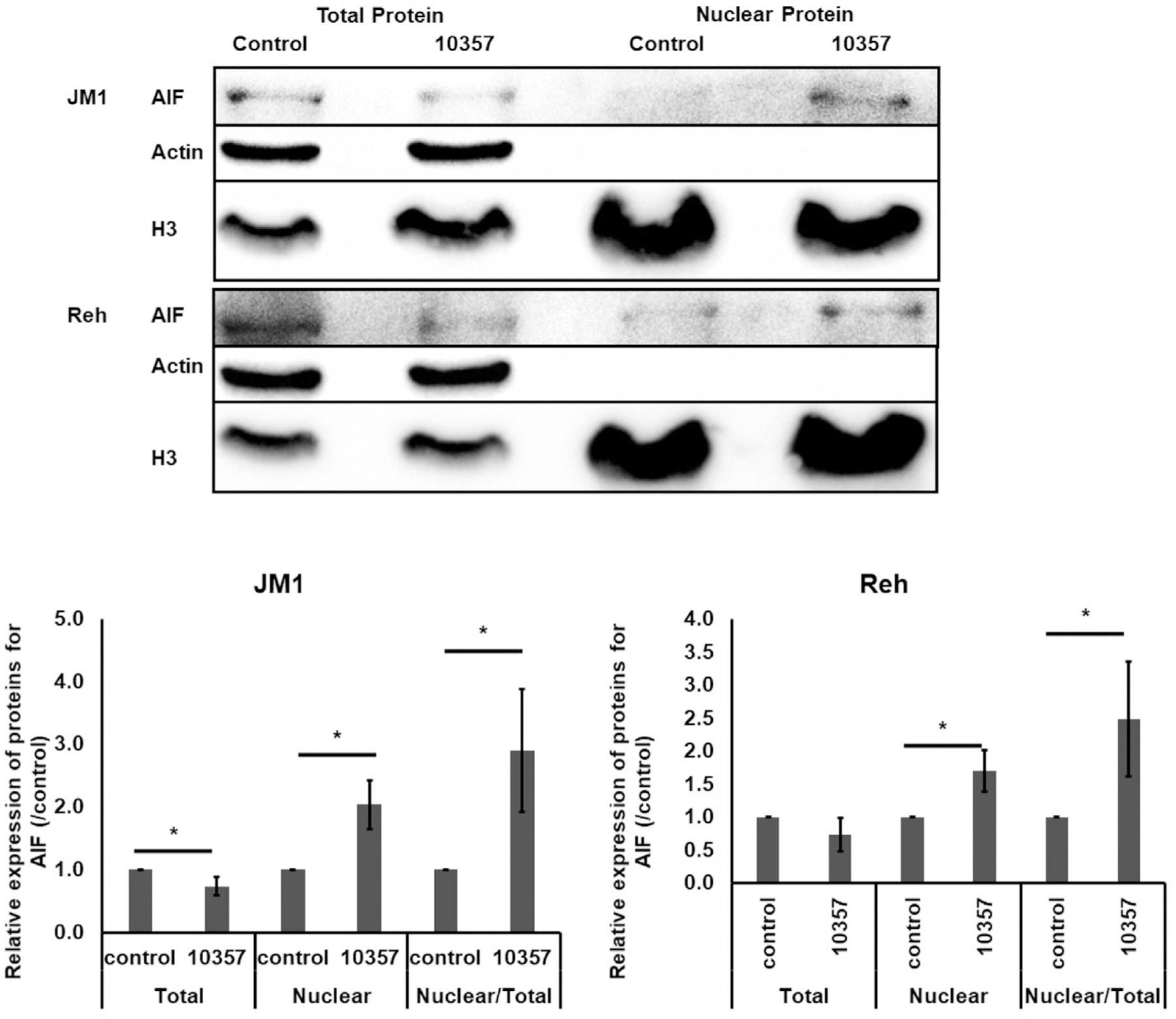
Cmpd10357 induces AIF translocation from the cytoplasm into the nucleus. Cells were treated with Cmpd10357 (JM1 22 *μ*M and Reh 26 *μ*M, n = 3) for 24 hours and fractionated into total protein and nuclear components. Fractions were immunoblotted for actin as a cytoplasmic marker, histone H3 as a nuclear marker, and AIF. Bands from the blot depicted in were digitally quantified, and the relative expression of AIF was plotted for each condition. AIF migrated into the nucleus 24 hours after exposure to Cmpd10357. Data are presented as the mean ± SD. *p < 0.05.

AIF is a mitochondrial protein, which is released and translocated to the nucleus upon exogeneous cellular stress, and is one of the programed cell death executioners that leads to cell death phenotypes, such as DNA fragmentation [9,10]. Cmpd10357 is shown to cause an AIF-mediated, nonapoptotic necrosis in glioma [5]. To determine whether Cmpd10357 contributes to AIF nuclear translocation in B-ALL cells, nuclear fractions were separated from total cell fractions, and AIF expression was analyzed by immunoblot analyses (Figure 3). The nuclear expression of AIF was significantly increased in both JM1 and Reh cells treated with Cmpd10357 compared with control cells after 24 hours of Cmpd10357 treatment. Altogether, these results suggest AIF-mediated, nonapoptotic necrosis [6,11] in B-ALL cells.

In this study, we report that Cmpd10357 demonstrates nonapoptotic cell death and AIF nuclear translocation in B-ALL cells. These results are consistent with earlier investigations in high-grade glioma showing that Cmpd10357 caused AIF release from mitochondria and nuclear translocation associated with AIF-mediated necrotic cell death [5,12].

Necrostatin-1 completely reversed the cytotoxicity of Cmpd10357 in JM1 and Reh cells (Figure 2B). RIPK1 is known as a key target molecule of Necrostatin-1 [13]. RIPK1 was reported to induce necroptosis via MLKL phosphorylation [14]. However, Necrosulfonamide, an MLKL inhibitor, did not alter the cytotoxicity of Cmpd10357 in Reh and JM1 cells (Figure 2C). These findings suggest that Cmpd10357 causes nonapoptotic cell death by a mechanism different from necroptosis in B-ALL cells.

Importantly, this nonapoptotic necrosis mechanism produced by Cmpd10357 is independent of cellular proliferation [6], thereby providing a rationale for combination with traditional ER stress and/or caspase-dependent drugs [15]. For example, poly(adenosine diphosphate ribose) polymerase (PARP) inhibitors, which are activated in multiple necrosis pathways, have recently shown efficacy in clinical trials to treat cancer in combination with chemotherapeutic agents [14]. Future work in this direction may involve both in vitro and in vivo studies of Cmpd10357 combined with established chemotherapeutic agents, such as etoposide or doxorubicin, which have been shown to be caspase-dependent, to evaluate the efficacy of a Cmpd10357 combination therapy [16,17].

## Supplementary Material

1 Supplemental Figure 1 **Cmpd10357 does not induce ER stress**. Cells were treated with 22 *μ*M Cmpd10357 (JM1 n=5, upper panel, Reh n=3–5, lower panel) for 6, 8 and 24 hours. No significant increase in expressions, except P-eIF2a in JM1 at 24 hours, is observed in Cmpd10357 treated cells. Actin was used for normalization of protein amount. All data are presented as the mean ± SD. * p<0.05.

## HIGHLIGHTS

- Cmpd10357 displays cytotoxicity in B-ALL cells and survival prolongation in a B-ALL mouse model.
- Cmpd10357’s cytotoxic mechanism seems independent of caspases and ER stress.
- Cmpd10357 demonstrates a nonapoptotic necrosis mechanism of cell death.
- Cmpd10357’s cytotoxic mechanism involves nuclear AIF translocation.
- This gives a rationale for combination chemotherapy with traditional apoptotic agents.
