# Supplementary material for: Cmpd10357 to treat B-cell acute lymphoblastic leukemia
Source: Exp Hematol. Author manuscript; Available in PMC 2023 Mar 23. (PMC10033359; doi:10.1016/j.exphem.2022.12.005)
Supplement: 1 — Supplemental Figure 1 Cmpd10357 does not induce ER stress. Cells were treated with 22 μM Cmpd10357 (JM1 n=5, upper panel, Reh n=3–5, lower panel) for 6, 8 and 24 hours. No significant increase in expressions, except P-eIF2a in JM1 at 24 hours, is observed in Cmpd10357 treated cells. Actin was used for normalization of protein amount. All data are presented as the mean ± SD. * p<0.05. [file NIHMS1869514-supplement-1.pdf]

Experimental Hematology
Volume 000

SUPPLEMENTAL METHODS

Cell Lines

JM1 and Reh cells were maintained in 75cm^2 plastic tissue culture-treated flasks (Corning Inc, New York, USA) at 37°C in a 5% CO_2 incubator. The JM1 cell line was grown in IMDM (Thermo Fisher Scientific) supplemented with 10% heat-inactivated fetal bovine serum (Thermo Fisher Scientific), 100U/mL penicillin and 100ug/mL streptomycin (Thermo Fisher Scientific), and 0.05mM 2-mercaptoethanol (Thermo Fisher Scientific). The Reh cell line was grown in RPMI 1640 (Thermo Fisher Scientific) supplemented with 10% heat-inactivated fetal bovine serum (Thermo Fisher Scientific), 100U/mL penicillin and 100ug/mL streptomycin (Thermo Fisher Scientific), 0.25% D-glucose (Sigma-Aldrich, St Louis, MO), 1mM sodium pyruvate (Thermo Fisher Scientific), and 10mM HEPES buffer (Thermo Fisher Scientific). Cells were manually washed and counted in PBS (Thermo Fisher Scientific) using trypan blue exclusion (0.04%, Sigma-Aldrich) and a hemocytometer. Cells were used within the first 25 passages and were maintained at their logarithmic phase of growth prior to each experiment.

MTS Assay

On the assay day (24 or 72 hours after treatment), 3-(4,5-dimethylthiazol-2-yl)-5-(3-carboxymethoxyphenyl)-2-(4-sulfophenyl)-2H-tetrazolium (MTS [Promega]) and N-methyl-phenazonium methosulfate (PMS [Sigma-Aldrich, Burlington, Massachusetts, USA]) were mixed at a 20:1 ratio and added to the cells for 2-4 hours. The absorbance was measured at 490nm using a microplate reader (Molecular Devices). The absolute optical densities were normalized to time-matched control cells and expressed as percent viabilities.

Immunoblot Analysis

Total protein and nuclear protein extracts were boiled and 10-30mg of protein was loaded on an 8−12% Tris-Glycine SDS-PAGE gel then transferred to a PVDF membrane using a semi-dry transfer system. Blots were blocked, incubated with primary antibodies overnight at 4°C, then incubated with secondary antibodies for 1 hour at room temperature. Primary antibodies used were anti-phospho-eIF2a (P-eIF2a, 1:1000), anti-ATF4 (1:1000), anti-CHOP (1:1000), anti-AIF (1:1000), anti-actin (1:20000), and anti-histone H3 (1:10000). Secondary antibodies used at 1:10,000 dilution were anti-rabbit HRP-linked and anti-mouse HRP-linked antibody. Anti-AIF antibody was purchased from Santa Cruz Biotechnology, Santa Cruz, California, USA while others were from Cell Signaling Technology, Danvers, Massachusetts, USA. Blots were washed, then incubated with SuperSignal™ West Femto Maximum Sensitivity Substrate (Thermo Fisher Scientific) for imaging. Bands were visualized using ChemiDoc MP Imaging System (Bio-Rad, Benicia, California, USA). Bands from the blot depicted were digitally quantified and the chemiluminescence intensities were normalized to actin or histone H3 as the internal controls for total cell fractions or nuclear fractions, respectively. The normalized intensities were then compared to time-matched control cells and expressed as relative protein expressions.

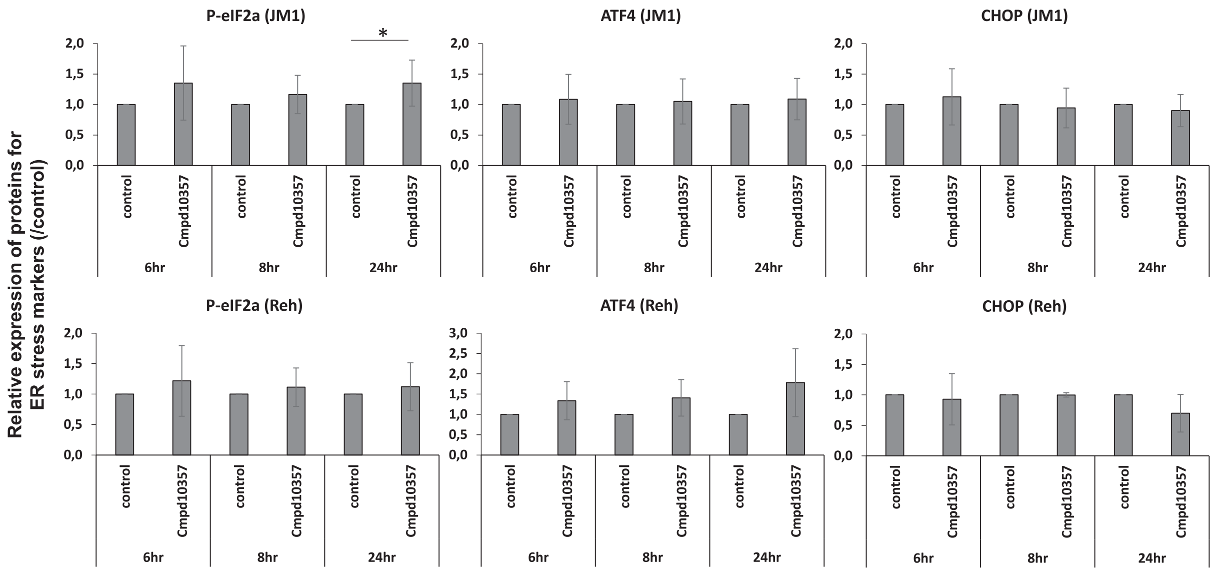

Supplemental Figure 1 Cmpd10357 does not induce ER stress. Cells were treated with 22 μM Cmpd10357 (JM1 n=5, upper panel, Reh n=3-5, lower panel) for 6, 8 and 24 hours. No significant increase in expressions, except P-eIF2a in JM1 at 24 hours, is observed in Cmpd10357 treated cells. Actin was used for normalization of protein amount. All data are presented as the mean ± SD. * $p<0.05$.
